# The IFN-γ-Inducible GTPase, Irga6, Protects Mice against *Toxoplasma gondii* but Not against *Plasmodium berghei* and Some Other Intracellular Pathogens

**DOI:** 10.1371/journal.pone.0020568

**Published:** 2011-06-17

**Authors:** Oliver Liesenfeld, Iana Parvanova, Jens Zerrahn, Seong-Ji Han, Frederik Heinrich, Melba Muñoz, Frank Kaiser, Toni Aebischer, Thorsten Buch, Ari Waisman, Gaby Reichmann, Olaf Utermöhlen, Esther von Stebut, Friederike D. von Loewenich, Christian Bogdan, Sabine Specht, Michael Saeftel, Achim Hoerauf, Maria M. Mota, Stephanie Könen-Waisman, Stefan H. E. Kaufmann, Jonathan C. Howard

**Affiliations:** 1 Institute of Microbiology and Hygiene, Charité Universitätsmedizin Berlin, Berlin, Germany; 2 Institute for Genetics, University of Cologne, Cologne, Germany; 3 Department of Immunology, Max Planck Institute for Infection Biology, Berlin, Germany; 4 Department of Molecular Biology, Max Planck Institute for Infection Biology, Berlin, Germany; 5 Institute of Medical Microbiology, University of Düsseldorf, Düsseldorf, Germany; 6 Institute of Medical Microbiology, Immunology and Hygiene, University of Cologne, Cologne, Germany; 7 Department of Dermatology, Johannes Gutenberg University, Mainz, Germany; 8 Department of Medical Microbiology and Hygiene, Institute of Medical Microbiology and Hygiene, University Hospital of Freiburg, Freiburg, Germany; 9 Microbiology Institute - Clinical Microbiology, Immunology and Hygiene, University Hospital of Erlangen, Erlangen, Germany; 10 Institute for Medical Microbiology, Immunology and Parasitology, University of Bonn, Bonn, Germany; 11 Malaria Unit, Instituto de Medicina Molecular, Faculty of Medicine, University of Lisbon, Lisbon, Portugal; Duke University Medical Center, United States of America

## Abstract

Clearance of infection with intracellular pathogens in mice involves interferon-regulated GTPases of the IRG protein family. Experiments with mice genetically deficient in members of this family such as Irgm1(LRG-47), Irgm3(IGTP), and Irgd(IRG-47) has revealed a critical role in microbial clearance, especially for *Toxoplasma gondii*. The *in vivo* role of another member of this family, Irga6 (IIGP, IIGP1) has been studied in less detail. We investigated the susceptibility of two independently generated mouse strains deficient in Irga6 to *in vivo* infection with *T. gondii*, *Mycobacterium tuberculosis*, *Leishmania mexicana*, *L. major, Listeria monocytogenes, Anaplasma phagocytophilum* and *Plasmodium berghei*. Compared with wild-type mice, mice deficient in Irga6 showed increased susceptibility to oral and intraperitoneal infection with *T. gondii* but not to infection with the other organisms. Surprisingly, infection of Irga6-deficient mice with the related apicomplexan parasite, *P. berghei*, did not result in increased replication in the liver stage and no Irga6 (or any other IRG protein) was detected at the parasitophorous vacuole membrane in IFN-γ-induced wild-type cells infected with *P. berghei in vitro*. Susceptibility to infection with *T. gondii* was associated with increased mortality and reduced time to death, increased numbers of inflammatory foci in the brains and elevated parasite loads in brains of infected Irga6-deficient mice. *In vitro*, Irga6-deficient macrophages and fibroblasts stimulated with IFN-γ were defective in controlling parasite replication. Taken together, our results implicate Irga6 in the control of infection with *T. gondii* and further highlight the importance of the IRG system for resistance to this pathogen.

## Introduction

Infection with the obligate intracellular pathogen *Toxoplasma gondii* causes disease in humans and animals [1], [2]. Following oral uptake, active invasion of host cells in the small intestine results in formation of parasitophorous vacuoles (PV) that resist lysosome fusion and thereby allow intracellular survival [3]. Following replication, dissemination, and initial immune activation, the parasite forms cysts in the central nervous system and skeletal muscle and persists lifelong. Whereas acute infection only rarely causes symptoms in man, severe immunosuppression (i.e., AIDS and transplantation) may result in reactivation of latent infection presenting as toxoplasmic encephalitis [1]. Furthermore, acute infection during pregnancy carries a high risk of transfer to the fetus and severe toxoplasmosis in the infant. Limitation of parasite replication and cyst formation relies on the development of a type 1 T helper (Th1) response involving secretion of IL-12 by dendritic cells [4] and interferon-gamma (IFN-γ) and tumor necrosis factor (TNF) by T cells and natural killer (NK) cells [5]. Murine models have been used extensively to study the immunopathogenesis of infection [5], [6]. IFN-γ has been identified as a key cytokine secreted mostly by CD4^+^ T cells and NK cells [7], [8]. The functions of IFN-γ include activation of macrophages and other antigen-presenting cells including astrocytes [9]–[11]. Mice on the C57BL/6 background (H-2^b^ haplotype) develop a fatal chronic progressive infection; pathologic changes include parasite-associated follicular infiltrations in the brain parenchyma and meninges similar to toxoplasmic encephalitis in AIDS patients [12], [13]. In contrast, mice on the BALB/c (H-2^d^) background and outbred strains of mice are resistant to the infection and do not develop symptoms of encephalitis [12].

Resistance against *T. gondii* in mice is partly dependent upon various members of the interferon-inducible 47 kDa GTPase family, recently designated the immunity-related GTPase (IRG) family [14], [15]. Twenty-three IRG genes have been identified in C57BL/6 mice [16]. Of those, the majority have been shown to be inducible by IFN-γ, including Irgm1 (formerly LRG-47), Irgm3 (formerly IGTP), Irgd (formerly IRG-47), Irgm2 (formerly GTPI), Irga6 (formerly IIGP), and Irgb6 (formerly TGTP) [16]–[21]. Targeted disruption of Irgm1 [22], Irgd [22] and Irgm3 [23] has revealed significant roles for each of these gene products in immunity *in viv*o against intracellular pathogens (reviewed in [24]). Irgm1 knockout mice were susceptible to a range of organisms including intracellular protozoa (*Leishmania major, Trypanosoma cruzi* and *T. gondii)* and intracellular bacteria (*Listeria monocytogenes, Salmonella typhimurium, Mycobacterium avium, M. tuberculosis* and *Chlamydia trachomatis*) (reviewed in [24]) though the basis for this dramatic loss of immune competence has recently been re-evaluated [25]. In contrast, mice lacking Irgd were susceptible to infection with *T. gondii* but resistant to *L. monocytogenes, S. typhimurium, M. tuberculosis*, and MCMV (reviewed in [24]). Irgm3 knockout mice were susceptible to infection with *T. gondii*, *L. major*, and *C. trachomatis* but showed resistance to *T. cruzi, L. monocytogenes, S. typhimurium, M. avium, M. tuberculosis* and MCMV (reviewed in [24]) The role of IIGP/Irga6 is less well documented. However, like many other IRG proteins Irga6 also accumulates rapidly on the parasitophorous vacuole membrane (PVM) surrounding intracellular parasites [26], [27]. Astrocytes isolated from the Cologne strain of Irga6-deficient mice described here had a partial loss of IFN-γ-mediated growth restriction of *T. gondii* [26], but no other details of the phenotype or of the gene disruption were given. Preliminary results from the same knock-out strain suggested no significant loss of *in vivo* resistance [14]. In the present paper we now provide full documentation for both this and an independently derived Irga6-deficient mouse strain from Berlin.

We show that both the Cologne and Berlin strains of Irga6-deficient mice have increased susceptibility to oral or intraperitoneal infection with *T. gondii.* Susceptibility to infection with *T. gondii* is associated with increased mortality, increased numbers of inflammatory foci in the brains and elevated parasite loads in organs of infected mice. Irga6-deficient macrophages stimulated with IFN-γ *in vitro* showed a partial defect in controlling parasite replication. Taken together, results of the present study add Irga6 to the list of IRG proteins whose loss in mice leads to increased susceptibility to *T. gondii*. Indeed, the significance of *T. gondii* as a major target of the IRG resistance system is emphasized by the lack of a detectable susceptibility phenotype of the Irga6-deficient animals for a long list of other intracellular pathogens, including the related apicomplexan parasite, *Plasmodium berghei*, which is assayed here for the first time in the context of IRG protein-mediated resistance.

## Materials and Methods

### Ethics statement

All animal experiments were conducted under the regulations governing animal experimentation in the relevant jurisdictions. Specific license numbers and authorities are as follows:

Aebischer: LaGeSo Berlin No. G 0170.01

Bogdan: Regierungspräsidium Freiburg No., 35-9185.81/G-03/70

Howard: LANOV Nordrhein-Westfalen No. 44.07.189

Liesenfeld: LaGeSo Berlin Nos. G 0258/04, 0113/06, 0114/06, 0109/08, 0146/10

Mota: All protocols were approved by the Animal Care Committee of the Instituto de Medicina Molecular, Lisbon, following Institutional, National, and European Union guidelines.

Reichmann: Düsseldorf No. 50.05-230-63/04

Specht: Bonn No. 50.203.2BN15,50/05

von Stebut: Landesuntersuchungsamt Rheinland-Pfalz No. 23 177/07/G08-1-010

Zerrahn: LaGeSo Berlin No. G0177-01

### Gene targeting and mice

Mice were generated at the Max Planck Institute for Infection Biology (MPIIB) in Berlin and independently with a different strategy at the Institute for Genetics (IG), University of Cologne. At MPIIB, the IRG locus was targeted in 129/OLA-derived E14 ES cells by replacement of the single coding exon of Irga6 with a neomycin resistance cassette. The deleted locus was backcrossed onto the 129/SvJ and C57BL/6 strains and bred to homozygosity. In Cologne, the Irga6 long coding exon was flanked by LoxP sites in Bruce 4 (C57BL/6) ES cells, and this exon was subsequently deleted in the germ-line by breeding mice carrying the floxed locus with Cre-deleter strain. The deleted locus was backcrossed to C57BL/6 and bred to homozygosity. See Figure S1 and Figure S2 for further details and verification of the two gene targeting strategies.

### Mice and infection

The homozygous line derived from the Berlin strategy is designated **B**-Irga6^−/−^ throughout the paper, and the line derived from the Cologne strategy **K**-Irga6^−/−^. When the genotype is not specified, mice from the two sources are referred to as **B**-strain and **K**-strain All mice were kept according to national guidelines for animal care in SPF animal facilities.

### *T. gondii*

Cysts of the type II ME49 or DX strains of *T. gondii*, avirulent in mice, were obtained from homogenized brains of mice intraperitoneally (i.p.) infected with 10 cysts for 2–3 months. 129Sv/J and C57BL/6 control mice and **B**-Irga6^−/−^ mice on the same backgrounds (laboratory of O. Liesenfeld) were infected perorally with 10 cysts in 0.3 ml phosphate buffered saline (PBS: pH 7.4) by gavage. **K**-C57BL/6 and **K**-Irga6^−/−^ mice were infected i.p. with bradyzoites isolated from 5 cysts (laboratory of G. Reichmann), and also according to the Berlin protocol (laboratory of O. Liesenfeld).

### *M. tuberculosis*

For infection with *M. tuberculosis*, **B**-strain mice (laboratory of J. Zerrahn) were aerosol-challenged with 100–200 colony-forming units (CFU) of *M. tuberculosis* H37Rv bacteria per lung using an aerosol chamber (Glas-Col) as described recently [28]. The inoculum was confirmed at day 1 post-infection (p.i.) by plating the lungs of control mice. Bacterial load per organ was analyzed by plating complete lungs and spleens of mice onto Middlebrook 7H11/ampicillin plates.

### *L. monocytogenes*

*L. monocytogenes* strain EGD bacteria were freshly prepared for infection from aliquots stored at −80°C. Aliquots were thawed and bacterial titers were determined by plating serial dilutions on TSB or blood agar plates. For intravenous (i.v.) infection, bacteria were injected in a volume of 200–500 µl phosphate buffered saline (PBS) into the lateral tail vein of **B** strain (laboratory of J. Zerrahn) or **K** strain mice (laboratory of O. Utermöhlen).

### *L. major* and *L. mexicana*

B-strain mice were infected subcutaneously with 2×10^6^ stationary phase promastigotes in 20 µl of PBS into the left hind footpads The inocula consisted of stationary phase promastigotes of *L. mexicana* (MNYC/BZ/62/M379) or *L. major* (WHOM/IR/-/173**).** Stationary phase *L. major* promastigotes were further enriched for metacyclic parasites by density centrifugation as described previously [29]. Inocula were opsonized before infection with 5% C5-deficient serum obtained from B10.D2/OsNj mice, by incubation at 37°C for 30 min (laboratory of T. Aebischer). Infective stage (metacyclic) promastigotes of *L. major* clone VI (MHOM/IL/80/Friedlin) were isolated from stationary culture by lack of agglutination with peanut agglutinin (Vector Laboratories, Burlingame, CA). K strain mice were infected by injection of 10 ml of PBS containing 2×10^5^ (high dose) or 10^3^ (physiologically relevant low dose infection) non-opsonised metacyclic promastigotes into the dorsal dermis of the left ears. Lesion volumes were measured weekly in 3 dimensions using a caliper, and are reported (in mm^3^) as ellipsoids [(a/2b/2c/2)4/3] [30] (laboratory of E. von Stebut).

### *A. phagocytophilum*

*A. phagocytophilum* strain Webster (formerly human granulocytic ehrlichiosis (HE) agent Webster strain) [31] was cultured in HL60 cells grown in RPMI 1640 medium supplemented with 1% fetal calf serum in 5% CO_2_ and used for mouse infection experiments as described previously [32]. Three **K** strain and three C57BL/6 mice per group were infected i.p. and sacrificed at days 3, 7, and 14 p.i. The bacterial load in blood, spleen, and lung was measured by quantitative PCR as copy number of *A. phagocytophilum* per copy number of mouse G6PDH [32]. The infectious dose was determined retrospectively and consisted of 1×10^5^ genome equivalents (laboratory of F. v. Loewenich and C. Bogdan).

### *Plasmodium berghei*

*P. berghei* ANKA was maintained by periodic passages through the vector *Anopheles stephensi*. For infection, sporozoites were isolated from salivary glands of mosquitoes fed at least 18 days earlier on mice containing circulating gametocytes (laboratories of A. Hoerauf and M. Mota). Mice were infected by intravenous (i.v.) injection of 20,000 *P. berghei* ANKA sporozoites. Parasite load in the liver was measured 40 h p.i. by qRT PCR as described previously [33].

Hepa1–6 cells growing on coverslips in 6-well plates were stimulated with 200 units of IFN-γ for 24 h before infection with 10,000 or 50,000 freshly isolated sporozoites per well (laboratory of S. Specht).

### Histology

To determine the extent of histological changes and the numbers of *T. gondii* cysts in brains and livers of mice, tissue samples were obtained at indicated time points after infection and immediately fixed in 5% formalin and embedded in paraffin. Sections (5 µm) were stained with hematoxylin and eosin and examined by light microscopy. Numbers of inflammatory areas were counted in 10 optical fields (200 magnification) in five adjacent sections each per mouse by blinded duplicate evaluation (S.H. and F.H.). Numbers of cysts were determined by immunohistology using *T. gondii*-antiserum in 10 optical fields (200 magnification) in five sections each per mouse. There were at least four mice per group. All experiments were repeated at least twice.

Immunofluorescence analysis of tissue culture cells infected with *P. berghei* was performed on glass cover slips essentially according to the protocol used earlier for *T. gondii* [26]. Irga6 was identified in IFN-γ-induced cells with rabbit anti-Irga6 antiserum 165 bleed 3 [34]. Sporozoites were identified microscopically by immunofluorescence with the mouse monoclonal antibody 2E6 specific for Hsp70 [35].

### Cytokine concentrations

Cytokine concentrations were determined in sera and supernatants of homogenized brain and liver samples obtained from *T. gondii*-infected mice. Homogenization was performed with two rough glass slides in 1 ml PBS. Homogenized organ samples were centrifuged at 12,000 rpm for 10 min at 4°C. The supernatants were harvested and stored at −80°C. Serum was obtained from blood samples by centrifugation at 12,000 rpm for 10 min at 4°C. Serum was stored at −80°C. IFN- and TNF concentrations were determined by ELISA (BD-Biosciences, San Diego, CA, USA). Cytokine concentrations were normalized to the whole protein concentration determined for each sample using the Roti-Nanoquant kit (Roth, Karlsruhe, Germany).

### Replication of *T. gondii* in primary bone marrow-derived macrophages (B)

Primary bone marrow cells were obtained from bones of 8-week-old mice. The femur and lower leg of mice were dissected and muscles removed. The bones were transferred to cold sterile PBS. The ends of the bone were removed with scissors and the bone marrow was flushed with a syringe containing cold medium (RPMI/FCS/Penicillin/Streptomycin). Bone marrow cells were pooled and centrifuged at 1,200 rpm for 10 min at 4°C. The supernatant was discarded and the cells were resuspended in 15 ml medium. Cells were plated at a concentration of 10^6^ cells/ml in 12-well-plates at 5×10^5^/well. Cells were differentiated to macrophages by addition of 500 µl macrophage colony stimulating factor (MCSF) in medium and cultivated at 37°C and 5% CO_2_ as previously described [36]. After 5 days the supernatant was exchanged with fresh MCSF in medium and cells were incubated for another 2 days under the same conditions. Differentiated macrophages were released by pipetting following incubation in PBS at 4°C and plated in 96-well plates at 10^5^ cells/well and stimulated with 0, 10, or 100 U/ml IFN-γ (Calbiochem, Schwalbach, Germany). After 24 h, cells were infected with tachyzoites of RH strain of *T. gondii* expressing green fluorescent protein (GFP) (a kind gift of D. Soldati-Favre, University of Geneva) at a multiplicity of infection (MOI) of 5∶1 (tachyzoites:cells). After 2 h incubation, extracellular tachyzoites were removed with the supernatant and cells were incubated with fresh medium for another 48 h. Cells were harvested with cold media and stained with a PE-tagged CD86-antibody (BD-Biosciences). The percentage of infected cells was determined by flow cytometry on a FACS Calibur (BD-Biosciences).

### Replication of *T. gondii* in mouse bone marrow-derived macrophages (K)

Primary bone marrow-derived macrophages were prepared essentially as above except that supernatant from L2 mouse fibroblasts was used as a source of MCSF. After induction of cells for 24 h with IFN-γ at several concentrations, cells were infected with different multiplicities of *T. gondii* strain ME49 tachyzoites freshly harvested from human foreskin fibroblast-cell cultures. Incorporation of ^3^H-uracil into replicating tachyzoites was measured essentially according to [27].

### Statistics

Comparison of variables, including numbers of inflammatory foci, numbers of microorganisms and cytokine concentrations, between individual groups of mice were determined using Student's t-test, Welsh's t-test or two-tailed Mann-Whitney test. Levels of significance for mortality in mice were determined using Fisher's exact test. Probability (P) values of <0.05 were considered significant.

## Results

### Course of infection with *L. mexicana, L. major, M. tuberculosis, L. monocytogenes, and A. phagocytophilum* in Irga6^−/−^ mice

**Figure 1:**
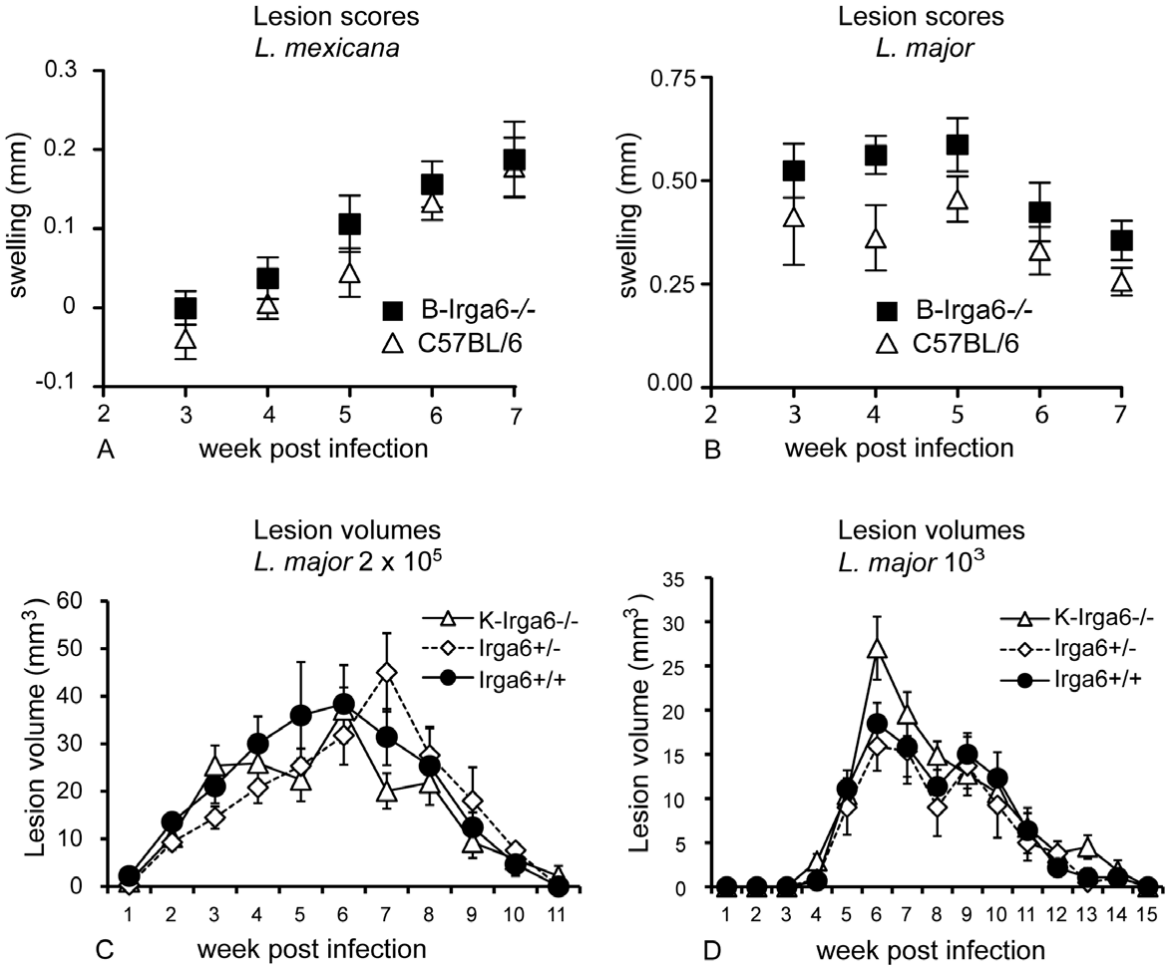
Role of Irga6 in infection with *L. mexicana* and *L. major.* B -Irga6^−/−^ and C57/BL6 control mice, in groups of 8 animals each, were infected by injection into the left hind footpad of *L. mexicana* (A) or *L. major* (B). Infection-induced swelling was determined weekly and values represent the mean differences in footpad thickness ± SEM. **K**-Irga6−/− and C57BL/6 control mice were injected with 2×10^5^ (C) or 10^3^ (D) *L. major* into the ear dermis. Lesion development was assessed weekly and expressed as ellipsoid (mean+/− SEM).

**Figure 2:**
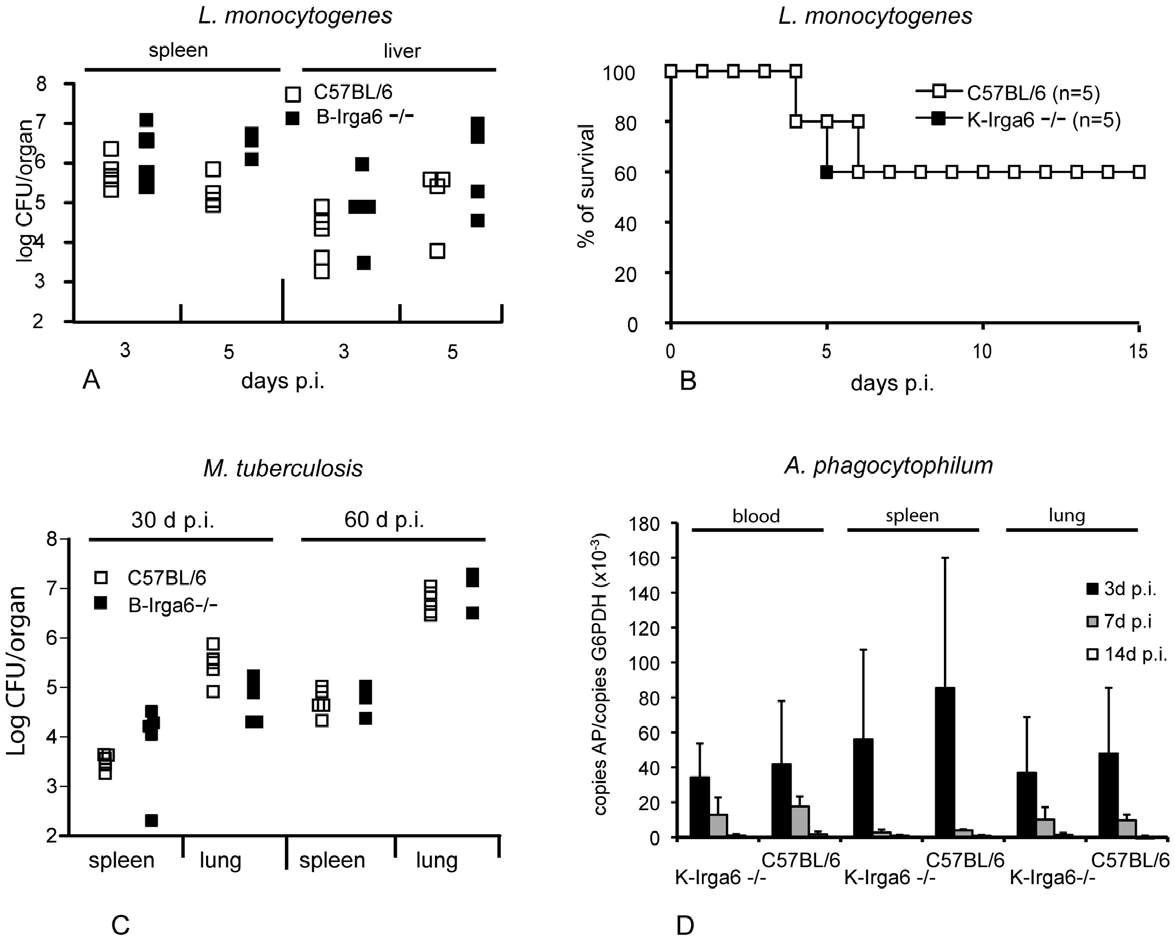
Role of Irga6 in infection with *M. tuberculosis, L. monocytogenes and A. phagocytophilum.* A: Infection of **B**-Irga6^−/−^ and wild type C57BL/6 mice with *L. monocytogenes*. CFU were measured in spleen and liver as described in Materials and Methods. B: Infection of **K**-Irga6^−/−^ and wild-type C57BL/6 mice with *L. monocytogenes.* Survival curves were measured as described in Materials and Methods C: Infection of **B**-Irga6^−/−^ mice with *M. tuberculosis*. CFU were measured in spleen and lung as described in Materials and Methods D: Infection of **K**-Irga6^−/−^ mice with *A. phagocytophilum.* The bacterial load was measured by quantitative PCR in blood, spleen and lung at the indicated day post infection (p.i.) as described in Materials and Methods. Means and SD from one experiment are shown.

Both Berlin (**B)** and Cologne **(K)** homozygous Irga6^−/−^ mutant mice were born at normal Mendelian ratios, were fertile, and did not exhibit any gross anatomical or behavioral abnormalities and the cellular composition and cellular phenotype of lymphoid organs was unaffected by the mutation (unpublished results). Groups of **B**-Irga6^−/−^ mice on a C57BL/6-background and wild-type littermates were infected with *L. mexicana* and *L. major* promastigotes into the hind footpad and infection-induced footpad swelling was recorded as a measure of disease severity. *L. mexicana*-infected animals developed slowly progressing lesions similar to, but marginally larger than, those of control mice over the first 7 weeks p.i. (Figure 1A); even after several months, no substantial differences were evident in lesion scores (not shown). *L. major*-infected mice developed transient self-healing infections again marginally larger in B-Irga6^−/−^ than in control mice (Figure 1B). Groups of **K**-Irga6^−/−^ mice on the C57BL/6 background were infected with *L. major* promastigotes in the dermis of the ear and lesion volumes were measured weekly in three dimensions with a caliper. One group of mice received a high promastigote dose (2×10^5^), the other group a low dose (10^3^) more closely resembling the natural infection. For both infection doses there was no significant difference between the K-Irga6^−/−^ mice and the heterozygous or homozygous wild-types (Figure 1C, D). The lesions appeared at the same time, developed to the same maximal size and healed in all infected animals. The tendency to larger lesions noted in the Berlin group was not repeated in the Cologne mice. Similarly, deficiency in Irga6 had no impact on the bacterial burden in organs from **B**-Irga6^−/−^ mice after i.v. infection with *L. monocytogenes* EGD (Figure 2A) and **K**-Irga6^−/−^ mice infected by i.p. injection of *L. monocytogenes* showed no excess mortality compared with wild-type C57BL/6 mice (Figure 2B). **B**-Irga6^−/−^mice showed no excess bacterial burden after aerosol infection with *M. tuberculosis* (Figure 2C). **K**-Irga6^−/−^ and wild-type C57BL/6 mice were infected i.p. with the intracellular bacterial pathogen, *A. phagocytophilum*, which replicates primarily in neutrophils. Organs harvested at three timepoints after infection were assayed by quantitative PCR for the bacterial *ankA* gene. The course of the self-resolving infection was identical in wild-type and **K**-Irga6^−/−^ mice (Figure 2D). Taken together, these results indicate that Irga6 is probably not significantly involved in resistance against *L. major* and *L. mexicana*, *L. monocytogenes*, *M. tuberculosis* and *A. phagocytophilum*.

### Course of infection with *T. gondii* in Irga6^−/−^ mice

**Figure 3:**
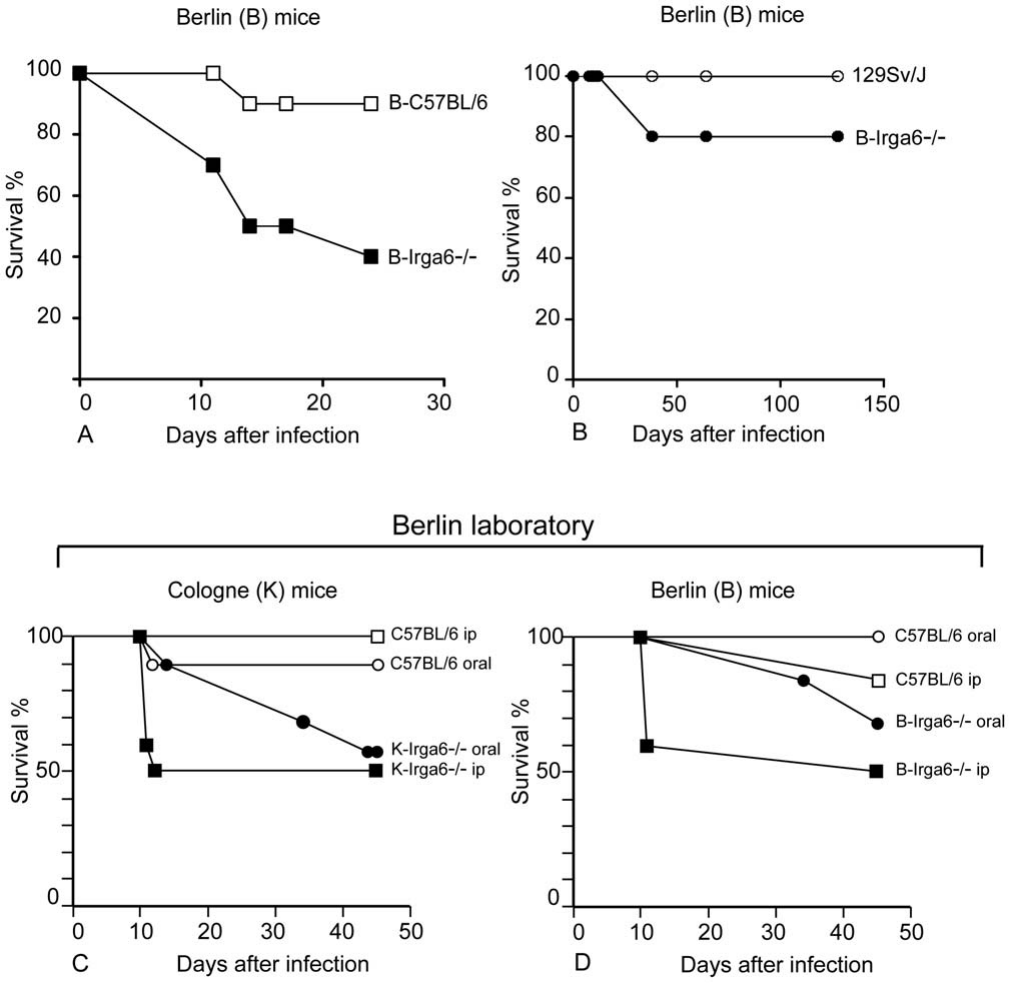
Survival of Irga6^−/−^ mice after oral and i.p. infection with *T. gondii.* Wild-type (open symbols) and **B-**Irga6^−/−^ mice (closed symbols) on the C57BL/6 (A) and 129Sv/J (B) background were infected orally with 10 cysts of the ME49 strain of *T. gondii*. There were at least 4 mice in each group. Results shown are from one representative experiment of three experiments performed. Survival and time to death of **K**-Irga6^−/−^ (C) and **B-**Irga6^−/−^ (D) mice (closed symbols) and their respective wild-type controls (open symbols) were compared following oral and i.p. infection with 10 cysts of the mouse-avirulent ME49 strain of *T. gondii*. All infections for this figure were performed in the Berlin laboratory.

We investigated the role of Irga6 in oral infection with the intracellular protozoon *T. gondii* in **B**-Irga6^−/−^ mice on the susceptible C57BL/6 and the resistant 129Sv/J backgrounds. Mice were orally infected with 10 cysts of *T. gondii.* The mortality of **B**-Irga6^−/−^ mice on the C57BL/6 background increased significantly (p<0.05) compared to wild-type mice (Figure 3A). A mortality of 60% in **B**-Irga6^−/−^ mice was observed by day 24 p.i. whereas wild-type mice showed a mortality of only 10% at the same time point. Both wild-type and B-Irga6^−/−^ mice on the 129Sv/J background were apparently somewhat more resistant to infection compared to mice on the C57BL/6 background. All wild-type 129Sv/J mice survived infection whereas the mortality rate was 10% in 129Sv/J Irga6^−/−^ mice during the observation period (up to day 150; Figure 3B). Taken together, these results indicated that Irga6 plays a role in resistance of C57BL/6 mice against oral infection with *T. gondii*.

A preliminary experiment on the susceptibility of **K**-Irga6^−/−^ mice on the C57BL/6 background to infection with the mouse-avirulent strains, ME49 and DX, yielded an insignificant excess mortality: of 22 wild-type and heterozygous animals, 1 died; of 9 Irga6^−/−^ homozygous animals, 2 died. These mortality numbers were originally interpreted conservatively to indicate that Irga6 deficiency did not lead to a significant susceptibility phenotype to *T. gondii* [37]. However the finding of susceptibility in the **B**-Irga6^−/−^ C57BL/6 strain mice (Figure 3A) contradicted this conclusion**.** To determine whether there was indeed a real difference between the two mutant strains, **B** and **K**-Irga6^−/−^ mice on C57BL/6 backgrounds were compared directly with wild-type C57BL/6 in the Berlin laboratory. All mice were infected with 10 cysts of the ME49 strain, either by the oral or intraperitoneal routes. The **B**- and **K**-Irga6^−/−^ strains behaved essentially identically in this experiment (Figure 3C, D). Both showed a weak but definite susceptibility phenotype relative to wild-type following infection by the oral route, and a somewhat greater susceptibility following infection by the intraperitoneal route. In both groups, 50% of the Irga6^−/−^ animals infected intraperitoneally died in the acute phase of the infection followed by prolonged survival. The experiment was terminated at 45 days.

**Figure 4:**
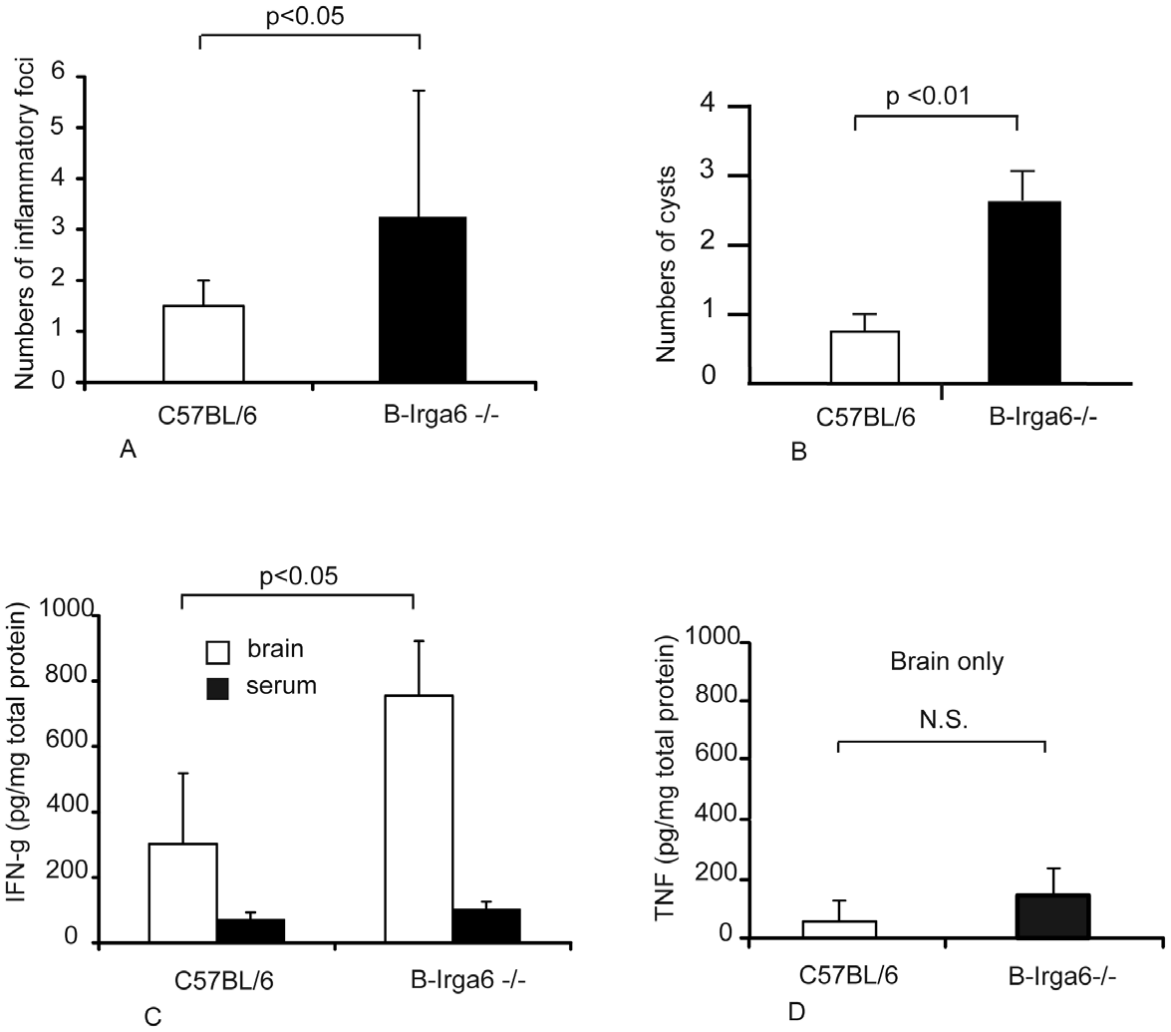
Inflammatory processes following *T. gondii* infection in the brains of B-Irga6 ^−/−^ and wild-type mice. (A) Inflammatory foci were detected histologically at 39 days after infection of **B**-Irga6^−/−^ with ME49 strain *T. gondii* as described in Materials and Methods. (B) Cysts were counted microscopically in brain homogenates obtained from infected mice 139 days after infection, as described in Materials and Methods. (C) IFN-γ and (D) TNF were determined by ELISA in brain homogenates and serum (C only) from **B**-Irga6^−/−^ mice at 29 days after infection. Means ± SD are presented from 4 individual mice; results are representative for 3 experiments performed.

To gain further insight into the increased susceptibility of Irga6^−/−^ mice to oral infection with *T. gondii*, we investigated inflammatory changes and parasite loads in brains of infected wild-type and **B**-Irga6^−/−^ C57BL/6 and 129/SvJ mice (Figure 4 and Figure S3). Four weeks after oral infection, the wild-type mice showed meningeal inflammation but only few inflammatory changes in the brain parenchyma compared with more pronounced meningeal and parenchymal inflammation (mostly mononuclear cells) in the Irga6^−/−^ mice (Figure 4A). Numbers of *T. gondii* cysts were significantly higher in the brains of **B**-Irga6^−/−^ mice compared to controls (Figure 4B). Taken together, these results suggest that mice deficient in Irga6 suffer from defective control of parasite replication resulting in increased inflammatory responses.

A limited histological analysis was also undertaken with the surviving mice from the experiment shown in Figure 3C and 3D above, in which **B**- and **K**-Irga6^−/−^ strain mice were compared directly for susceptibility to *T. gondii* infection. Mean inflammatory scores (considering meningeal inflammation and parenchymal foci of inflammation) [38] were more pronounced in Irga6^−/−^ mice compared to wild-type mice euthanized at day 45 p.i. regardless of the route of infection and the origin of mice (data not shown).

IFN-γ is the key cytokine for orchestration of immunity against infection with *T. gondii* [8] and also controls the induction of IRG proteins [39]. Hence, we determined concentrations of IFN-γ in brain homogenates and serum obtained from *T. gondii*-infected wild-type C57BL/6 and **B**-Irga6^−/−^ mice. Mean concentrations of IFN-γ were significantly elevated in brains but not in serum in Irga6^−/−^ compared to wild-type mice (Figure 4C). In contrast, the concentration of TNF did not differ significantly in brains in these animals (Figure 4D). These results indicate that local IFN-γ production is increased in Irga6^−/−^ mice, presumably due to the cellular reaction against increased parasite loads and that the phenotype of the Irga6^−/−^ mice is not due to a lack of IFN-γ.

### Irga6 contributes to the control of *T. gondii* replication in IFN-γ-stimulated macrophages

**Figure 5:**
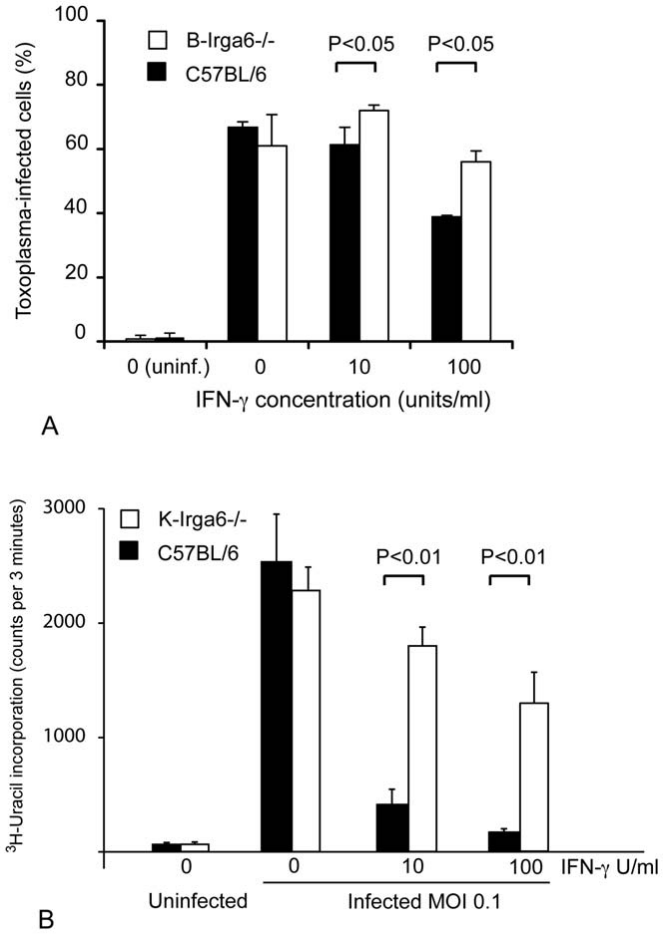
IFN-γ-dependent control of replication of *T. gondii* in bone-marrow-derived macrophages from wild-type and Irga6^−/−^ mice. (A) **B**-Irga6^−/−^ and wild type mice: Bone-marrow derived macrophages were pre-treated with IFN-γ at indicated doses and infected with GFP-expressing parasites of the mouse-virulent RH strain of *T. gondii* at a parasite-cell ratio of 5∶1. At 48 h post infection, percentages of infected CD86-positive cells were determined by flow cytometry. At zero infection the data record fluctuations around background. Data shown are results ± SD representative of 2 experiments performed. * p<0.05. A significant loss of IFN-dependent control was seen in the Irga6-deficient cells (open columns). (B): **K**-Irga6^−/−^ and wild-type C57BL/6 mice: bone-marrow derived macrophages were induced for 24 h with 10 units or 100 units IFN-γ and infected at MOI 0.1 with mouse-avirulent ME49-strain *T. gondii*. Proliferation of *T. gondii* was measured by incorporation of ^3^H-uracil between 48 and 72 h after infection. A highly significant loss of IFN-γ-dependent control was seen in the Irga6-deficient cells (white columns) at both IFN-γ concentrations.

To determine whether Irga6 is involved in control of parasite replication, bone marrow-derived macrophages (BMM) from **B**-Irga6^−/−^ mice were stimulated with IFN-γ and infected with RH tachyzoites. BMM from wild-type and Irga6^−/−^ mice could both be stimulated with IFN-γ to block the replication of *T. gondii* in a concentration-dependent manner. The replication block was not complete but percentages of infected cells were significantly higher in Irga6^−/−^ than in wild-type BMM following stimulation with 10 and 100 IFN-γ U/ml (Figure 5A). Similar experiments were performed with BMM from **K**-Irga6^−/−^ mice, in which replication of mouse-avirulent ME49 strain *T. gondii* in IFN-γ-stimulated cells was monitored by incorporation of ^3^H-uracil. Again a highly significant loss of IFN-γ-dependent control of parasite proliferation was seen in the K-Irga6^−/−^ cells compared with the K-C57BL/6 wild-type cells (Figure 5B). Similar results were reported earlier in K-Irga6^−/−^ embryonic astrocytes [26] and in mouse embryonic fibroblasts (unpublished). The greater impairment in resistance seen in Figure 5B than in Figure 5A is probably due to the lower MOI used in the experiment in Figure 5B (unpublished results). These results indicate that IIGP/Irga6 contributes to IFN-γ-mediated cell-autonomous control of *T. gondii*, and are consistent with the significant but limited susceptibility to low-dose infection with mouse-avirulent *T. gondii in vivo* shown above (Figure 3).

### *P. berghei* load in liver of wild-type and K-Irga6^−/−^ mice

**Figure 6:**
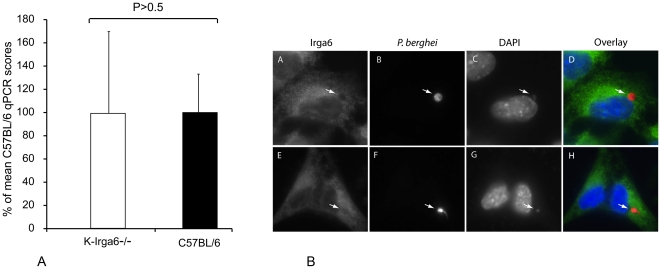
The IRG system does not interact with *P. berghei*. (A) No effect of Irga6 deficiency on *P. berghei* replication in the liver. K-Irga6^−/−^ and wild-type C57BL/6 mice were infected intravenously with 20,000 fresh mosquito-derived *P. berghei* ANKA sporozoites. 40 hours after infection DNA was prepared from livers for qPCR with primers specific for the *P. berghei* 18S gene as described in Materials and Methods. 14 mice from each strain were assayed. (B) Irga6 does not accumulate on the *P. berghei* parasitophorous vacuole membrane. Hep1-6 cells were induced with 200 units of IFN-γ. After 24 h, cells were infected with fresh mosquito-derived sporozoites of *P. berghei* ANKA, fixed 6 h later and stained with a rabbit antiserum (165) against Irga6 (green). 2 infected cells are illustrated (panels A-D and E–H). The sporozoites (white arrows) were identified with a mouse antibody (2E6) directed against a *P. berghei* hsp70 (red). DAPI was used to identify nuclei of both the Hep1-6 and *P. berghei.*

Irga6 is expressed constitutively in hepatocytes and is also strongly induced in liver by IFN-γ [39], [40]. This distribution hinted that Irga6 might act preferentially in the liver, and therefore be of special relevance in resistance to the related parasite *P. berghei,* which infects hepatocytes before infecting red blood cells and causing malaria [41] We therefore assessed a possible effect of Irga6 on *P. berghei* resistance by measuring parasite load during the early replication phase in the liver using quantitative PCR for PbA 18S ribosomal RNA following infection of wild type C57BL/6 and K-Irga6^−**/**−^ mice with large doses of sporozoites from the ANKA strain. No significant difference was detected over a total of 14 animals per genotype in three experiments (Figure 6A).

### Failure of IRG proteins to accumulate on *P. berghei* parasitophorous vacuole

If IRG proteins contribute to resistance against *P. berghei* in the same way as they do against *T. gondii*, it seemed reasonable to look for loading of Irga6 onto the PVM of *P. berghei* sporozoites infecting IFN-γ-induced liver cells. We looked with IRG-specific antibody reagents for accumulation of Irga6 and other IRG proteins at the *P. berghei* sporozoite parasitophorous vacuole in IFN-γ-induced Hep1-6 liver-derived cells infected with fresh, mosquito-derived *P. berghei* (ANKA) sporozoites. No accumulation of Irga6 (Figure 6B) or of any other IRG protein (not shown) was detected at the *P. berghei* PV. The same result was found in several other cell lines including primary C57BL/6 MEFs (not shown). We tentatively conclude that the IFN-γ-inducible IRG system does not play a significant role in resistance to *P. berghei* in the C57BL/6 mouse.

## Discussion

The present study shows that deficiency of Irga6/IIGP impairs survival of mice whether orally or peritoneally infected with *T. gondii* (Figure 3). The inflammatory response in brains of Irga6^−/−^ mice was significantly increased compared to wild-type controls, indicated by increased numbers of inflammatory foci and *T. gondii* cysts as well as higher levels of IFN-γ (Figure 4). By these criteria, susceptibility to *T. gondii* was more pronounced in Irga6^−/−^ mice on a C57/BL6 than on a 129Sv/J background. These results, accumulated from experiments based on two independent gene knock-outs prepared in different laboratories (Cologne, Germany, **K**, and Berlin, Germany, **B**) contradict an earlier suggestion [14], based on preliminary data for one of the two knock-out strains, that normal control of mouse-avirulent *T. gondii* infection in mice is independent of Irga6. In addition, analysis of BMM from both Irga6-deficient strains revealed an impaired IFN-γ-mediated control of *T. gondii* growth *in vitro*. These data can be added to the results already reported, of a smaller but clear deficit in control of *T. gondii* replication in IFN-γ-treated astrocytes from Irga6-deficient C57BL/6 mice [26]. Thus, Irga6/IIGP now clearly belongs to the list of IRG proteins that contribute to defence against *T. gondii in vivo* and *in vitro.*

In contrast, we found that deficiency of Irga6 did not influence survival or pathogen load in mice upon infection with several other intracellular pathogens including *M. tuberculosis*, *L. monocytogenes* and *A. phagocytophilum*. Apparently slightly reduced resistance was found to the two *Leishmania* species, *L. major* and *L. mexicana*, assayed on the **B**-Irga6^−/−^ mice (Figure 1A, B). If confirmed, this result would be consistent with a report of increased mortality to *Leishmania* in mice deficient in another IRG protein, Irgm3 [15]. However no equivalent trend towards susceptibility to *L. major* was apparent in **K**-Irga6^−/−^ mice (Figure 1C, D). Further experimentation will be required to find out whether this difference is merely a statistical accident. The association of IRG proteins with the *Leishmania* parasitophorous vacuole is presently under investigation. It is unlikely that IRG proteins can contribute to resistance against *Leishmania* without associating with the parasitophorous vacuole. The situation with *Chlamydia trachomatis* L2 strain is controversial. Irga6-deficient C57BL/6 mice of the Cologne strain have been shown clearly to have normal resistance to this pathogen both *in vivo* and in IFN-γ-induced embryonic fibroblasts *in vitro* [42]. However an earlier report exploited an RNAi procedure *in vitro* to indicate that Irga6 may play a role in resistance of IFN-γ-induced mouse cells to this strain [43] and it was also reported recently that IFN-γ-induced Irga6-deficient C57BL/6 embryonic fibroblasts from the Berlin strain lose resistance to *C. trachomatis* L2 [44]. These discrepant results have been obtained in different laboratories using different techniques and will need to be reconciled.

For the first time, we have also explored the possible contribution of IRG proteins to resistance to a malarial pathogen, *P. berghei*, in mice. In view of the taxonomic affinity between *Toxoplasma* and *Plasmodium* it was a reasonable expectation that IRG proteins would behave in a similar manner towards both apicomplexan parasites. However, the results do not support this expectation. Since IRG proteins function cell-autonomously in the cytoplasm, a reasonable assumption would be that any resistance mediated by IRG proteins against *Plasmodium* would be initiated during the intracellular replicative phase in hepatocytes. In the case of Irga6, this seemed especially plausible in view of the distinctive high constitutive expression of this IRG protein in the liver driven by a dedicated hepatocyte-specific promoter [40], and the extreme inducibility of liver Irga6 to IFN-γ [19], [40]. However, quantitative PCR failed to find any tendency towards higher parasite numbers in livers from Irga6-deficient than from wild-type C57BL/6 mice (Figure 6A). Lastly, and perhaps most definitively, no IRG proteins were found associated with the PV of *P. berghei* infecting several different IFN-γ-induced cell lines of liver and non-liver origin. This was the case not only for Irga6 (Figure 6B), but also for Irgb6, Irgd, Irgm1 and Irgm3 (data not shown). Preparations were examined throughout the infection from the first 30 minutes, with elongated sporozoites, to 12 hours after infection immediately after the beginning of replication. At no time was IRG protein loading observed. Since the resistance mechanism of IRG proteins for *T. gondii* entails loading onto the PVM [26] followed by disruption of the vacuolar membrane [26], [45] and death of the parasite [45] it seems clear that absence of IRG loading onto *P. berghei* vacuoles implies lack of a resistance function of IRG proteins for this parasite. For the moment, therefore, we consider that IRG proteins in general, and Irga6 in particular, are not involved in resistance of mice against *P. berghei*. Since pathogen-derived mechanisms from both *T. gondii* [27], and *Chlamydia muridarum* [42] can antagonise IRG protein function and reduce or eliminate their loading onto pathogen-containing vacuoles, a similar effect may also be at work in *P. berghei* infection of mice. In the absence of any IRG protein loading, such a virulence factor could operate freely in the cytosol to inactivate IRG proteins before they access the vacuolar membrane. Alternatively, the result could be due to expression of a factor at the vacuolar membrane that repels IRG protein loading. However, the principles of IRG protein loading onto pathogen vacuoles have not yet been elucidated. Despite many apparent similarities between the parasitophorous vacuoles of *T. gondii* and *P. berghei* there may yet be structural differences relevant to the IRG loading mechanism. Whether these constitute a virulence mechanism in *P. berghei* against the IRG system will depend on the ecological relationship between *P. berghei* and its natural hosts.

Including Irga6, four mouse IRG genes have now been disrupted and all have resulted in a more or less dramatic susceptibility to infection with *T. gondii.* With mice on a mixed C57BL/6 129Sv background 100% death within 14 days was seen after i.p. infection with 20 cysts of the mouse-avirulent ME49 strain *T. gondii* in Irgm3^−/−^ and Irgm1^−/−^ mice [23], [49]. Approximately 30% of Irgd^−/−^ mice died during the acute stage of infection and mortality increased to 70% by day 60 p.i. [49]. The intermediate acute susceptibility of Irga6^−/−^ mice against infection with *T. gondii* observed in susceptible C57BL/6 and resistant 129Sv mice in the present study more closely resembles the intermediate acute susceptibility to infection with *T. gondii* observed in Irgd^−/−^ mice [49], but without the later, progressive mortality seen in the Irgd knock-out on a different genetic background. The family of IRG proteins has been divided structurally and functionally into a regulatory subgroup, the GMS proteins, and an effector subgroup, the GKS proteins [39], [50]. For the moment, the data support the view that mice with a loss of individual regulatory (GMS) GTPases (Irgm1 and Irgm3) have stronger phenotypes than those with a loss of individual effector (GKS) GTPases (Irgd and Irga6). Since the GMS proteins probably regulate all the effector GTPases [50], the stronger phenotypes with loss of Irgm1 and Irgm3 could perhaps be anticipated.

The non-redundant requirement for at least four members of the IRG family, now including Irga6, confirms the complex integrated activity of the IRG system in organising cell-autonomous resistance to *T. gondii*. The regulatory role of the three GMS proteins on the GKS effector proteins is one part of the story [50], while the cooperative binding interactions of multiple effector proteins on the PV, leading ultimately to its disruption [27] is another. Through results on Irgm1-deficient mice the IRG system has been implicated in resistance to a number of parasites in addition to chlamydia and *T. gondii*. However it is likely that such results for pathogens other than *C. trachomatis* and *T. gondii* can all be attributed to a general immune deficiency caused indirectly by loss of Irgm1, and not to loss of a direct resistance action by Irgm1 itself [25]. It is indeed remarkable that the complex IRG system is so important for mice in resistance to only a few pathogens. It is also remarkable that the entire system has been lost in the primate lineage leading to man [16]. However, in view of the irreplaceable status of the mouse as the model of choice in experimental studies of immunity, it is essential to understand the differences as well as the similarities between the mouse and human immune systems [51].

## Supporting Information

Figure S1 **Generation of Irga6/IIGP gene deletion in Berlin.** In the Max Planck Institute for Infection Biology, Berlin, Irga6/IIGP was originally cloned by suppression subtractive hybridization from splenocytes isolated from *L. monocytogenes*-infected C57BL/6 mice [52]. The following strategy was employed to generate a deletion of the Irga6/IIGP gene (Figure S1). LAWRIST7-based cosmids MPMGc121F04152 and MPMGc121E14552 from murine genomic 129/Ola cosmid library (Deutsches Ressourcenzentrum fuer Genomforschung RZPD, Berlin, Germany) were identified as Irga6/IIGP positive by PCR and confirmed by sequencing. A 4684 bp EcoRI/PvuII fragment, immediately upstream of the single coding exon of the Irga6 gene, was cloned in front of a neomycin resistance cassette within a pBluescript vector. A 590 bp PCR-amplified Irga6 fragment immediately downstream of the Irga6 coding sequence was cloned behind the selection cassette followed by a herpes simplex virus thymidine kinase cassette (Figure S1A). The linearised targeting vector was electroporated into E14.1 ES cells [53]. Homologous recombinants were detected by PCR and confirmed by Southern blot hybridization with 5′ or 3′ flanking probes. Single integration was verified by probing the Southern blots with the neomycin resistance cassette. Correctly targeted ES cell clones were injected into C57BL/6 blastocysts and transferred to foster mothers. Chimeric progeny were backcrossed either to C57BL/6 or 129Sv/J mice, and germ line transmission of the targeted allele was confirmed by Southern blot. The mice used in these experiments were backcrossed to the C57BL/6 background for at least 7 generations or to the 129/SvJ background for at least 4 generations. Genotyping for the Irga6 mutation was performed by PCR with the following primers: 5′- CTGCTGACCTAGTGAATATCATC -3′ (Irga6 forward), 5′- CGCCTTCTTGACGAGTTCTTCTG (Neomycin forward), and 5′- AATGTGGATACATAATCAGTAAAGG -3′(Irga6 reverse). The endogenous, non-mutated locus gave rise to a 527 bp fragment, while the targeted locus produced an 810 bp fragment (Figure S1B). Generation of bone marrow-derived macrophages, stimulation with IFN-γ, and analysis of Irga6 deficiency by Western Blot with mAb 10E7 (Figure S1C). were performed as described previously PCR primers for generation of the short-arm probe (italic: Irga6-specific portion) were: 5′-ACGCGAG*GGTGAGTCCCAATGATCAGG* and 5′-ACGCGAG*AGAGGCTGCATTGGCTGTGGT* . PCR primers for generation of the long-arm probe were: CTTGGCTAAGGGTAAGGCCT and AGAGGCCCTCTGCCTTAGC.
(TIF)

Figure S2 **Generation of Irga6/IIGP gene deletion in Cologne.** At the Institute for Genetics in Cologne, Irga6/IIGP was originally cloned from IFNγ-induced C57BL/6 mouse embryonic fibroblasts by suppression subtractive hybridization [39]. The following strategy was employed to generate a deletion of the Irga6/IIGP gene. A 4kb BamH1 fragment, containing the whole of the coding exon 2 of Irga6 with about 2 kb of 5′ intronic sequence and about 1 kb of 3′-untranslated sequence, was subcloned from the C57BL/6 strain derived genomic BAC clone RP23-19A12 into the blunted Sal1 site of the targeting vector, pEasyFlox [54] 5′ and 3′ homology arms were generated by PCR amplification from BAC sublones. Additional restriction sites were added with the primers to enable the 5′ and 3′ homology arms to be cloned into the Xho1 and Not1 sites of pEasyFlox respectively. The completed targeting construct (Figure S2A) was linearised and transfected into Bruce4 ES cells derived from C57BL/6 Thy1.1 mice [55]. Of 350 G418 and gancyclovir-resistant colonies, 3 were identified as homologous integrants, of which one was probably partial. The remaining two clones, 1B10 and 3A3 were injected into CB20 blastocysts and high-level chimeras generated. Two chimeras derived from clone 1B10 transmitted the mutant allele to 100% of progeny. A completely Irga6-deficient mouse was generated by crossing germ-line-transmitting offspring to the C57BL/6 Cre-deleter mouse [56] Positive heterozygous progeny were intercrossed to generate homozygous Irga6-deficient progeny. All expected genotypes were generated in mendelian ratios, assayed by Southern blotting of tail-tip DNA. Embryonic fibroblasts prepared from the homozygous line of Irga6-deficient mice showed no signal in Western blot with rabbit anti-Irga6 serum 24 hr after induction with 100 U/ml IFN-γ (Figure S2B).
(TIF)

Figure S3 **Histological changes in brains of wildtype and B-Irga6-deficient mice following infection with** ***T. gondii*** **.** Histological changes in brains of mice infected orally with 10 cysts of the ME49 strain of *T. gondii*. At 29 (C57BL/6-background, A, B) and 139 (129Sv/J- background, E, F) days post infection brains of mice were obtained, sections prepared, and stained with H&E. Arrows in A, B, E, and F indicate meninges; arrowheads indicate areas of focal inflammation. To visualize parasites, sections of brains obtained at day 29 post infection were stained with an anti-*T. gondii* serum (C,D); *T. gondii* cysts are indicated by arrows and parasitophorous vacuoles are indicated by arrowheads.
(TIF)

## References

[1] Montoya JG, Liesenfeld O (2004). Toxoplasmosis.. Lancet.

[2] Tenter AM, Heckeroth AR, Weiss LM (2000). *Toxoplasma gondii*: from animals to humans.. Int J Parasitol.

[3] Sinai AP, Joiner KA (1997). Safe haven: the cell biology of nonfusogenic pathogen vacuoles.. Annu Rev Microbiol.

[4] Liu CH, Fan YT, Dias A, Esper L, Corn RA (2006). Cutting edge: dendritic cells are essential for *in vivo* IL-12 production and development of resistance against *Toxoplasma gondii* infection in mice.. J Immunol.

[5] Denkers EY, Gazzinelli RT (1998). Regulation and function of T-cell-mediated immunity during *Toxoplasma gondii* infection.. Clin Microbiol Rev.

[6] Suzuki Y (2002). Host resistance in the brain against *Toxoplasma gondii*.. J Infect Dis.

[7] Suzuki Y, Conley FK, Remington JS (1989). Importance of endogenous IFN-gamma for prevention of toxoplasmic encephalitis in mice.. J Immunol.

[8] Suzuki Y, Orellana MA, Schreiber RD, Remington JS (1988). Interferon-gamma: the major mediator of resistance against *Toxoplasma gondii*.. Science.

[9] Aschner M (1998). Immune and inflammatory responses in the CNS: modulation by astrocytes.. Toxicol Lett.

[10] Fagard R, Van Tan H, Creuzet C, Pelloux H (1999). Differential development of *Toxoplasma gondii* in neural cells.. Parasitol Today.

[11] Stark GR, Kerr IM, Williams BR, Silverman RH, Schreiber RD (1998). How cells respond to interferons.. Annu Rev Biochem.

[12] Kang H, Liesenfeld O, Remington JS, Claflin J, Wang X (2003). TCR V beta 8+ T cells prevent development of toxoplasmic encephalitis in BALB/c mice genetically resistant to the disease.. J Immunol.

[13] Suzuki Y (2002). Immunopathogenesis of cerebral toxoplasmosis.. J Infect Dis.

[14] Martens S, Howard J (2006). The interferon-inducible GTPases.. Annu Rev Cell Dev Biol.

[15] Taylor GA (2007). IRG proteins: key mediators of interferon-regulated host resistance to intracellular pathogens.. Cell Microbiol.

[16] Bekpen C, Hunn JP, Rohde C, Parvanova I, Guethlein L (2005). The interferon-inducible p47 (IRG) GTPases in vertebrates: loss of the cell autonomous resistance mechanism in the human lineage.. Genome Biol.

[17] Carlow DA, Marth J, Clark-Lewis I, Teh HS (1995). Isolation of a gene encoding a developmentally regulated T cell-specific protein with a guanine nucleotide triphosphate-binding motif.. J Immunol.

[18] Gilly M, Wall R (1992). The IRG-47 gene is IFN-gamma induced in B cells and encodes a protein with GTP-binding motifs.. J Immunol.

[19] Boehm U, Guethlein L, Klamp T, Ozbek K, Schaub A (1998). Two families of GTPases dominate the complex cellular response to interferon-gamma.. J Immunol.

[20] Sorace JM, Johnson RJ, Howard DL, Drysdale BE (1995). Identification of an endotoxin and IFN-inducible cDNA: possible identification of a novel protein family.. J Leukoc Biol.

[21] Taylor GA, Jeffers M, Largaespada DA, Jenkins NA, Copeland NG (1996). Identification of a novel GTPase, the inducibly expressed GTPase, that accumulates in response to interferon gamma.. J Biol Chem.

[22] Collazo CM, Yap GS, Hieny S, Caspar P, Feng CG (2002). The function of gamma interferon-inducible GTP-binding protein IGTP in host resistance to *Toxoplasma gondii* is Stat1 dependent and requires expression in both hematopoietic and nonhematopoietic cellular compartments.. Infect Immun.

[23] Taylor GA, Collazo CM, Yap GS, Nguyen K, Gregorio TA (2000). Pathogen-specific loss of host resistance in mice lacking the IFN-gamma-inducible gene IGTP.. Proc Natl Acad Sci U S A.

[24] Hunn JP, Feng CG, Sher A, Howard JC (2011). The Immunity-Related GTPases in mammals - a fast evolving cell-autonomous resistance system against intracellular pathogens.. Mammalian Genome.

[25] Hunn JP, Howard JC (2010). The Mouse Resistance Protein Irgm1 (LRG-47): A regulator or an effector of pathogen defense?. PLoS Pathog.

[26] Martens S, Parvanova I, Zerrahn J, Griffiths G, Schell G (2005). Disruption of *Toxoplasma gondii* parasitophorous vacuoles by the mouse p47-resistance GTPases.. PLoS Pathog.

[27] Khaminets A, Hunn JP, Konen-Waisman S, Zhao YO, Preukschat D (2010). Coordinated loading of IRG resistance GTPases on to the *Toxoplasma gondii* parasitophorous vacuole.. Cell Microbiol.

[28] Mollenkopf HJ, Hahnke K, Kaufmann SH (2006). Transcriptional responses in mouse lungs induced by vaccination with *Mycobacterium bovis* BCG and infection with *Mycobacterium tuberculosis*.. Microbes Infect.

[29] Spath GF, Beverley SM (2001). A lipophosphoglycan-independent method for isolation of infective *Leishmania* metacyclic promastigotes by density gradient centrifugation.. Exp Parasitol.

[30] Von Stebut E, Ehrchen JM, Belkaid Y, Kostka SL, Molle K (2003). Interleukin 1alpha promotes Th1 differentiation and inhibits disease progression in *Leishmania* major-susceptible BALB/c mice.. J Exp Med.

[31] Asanovich KM, Bakken JS, Madigan JE, Aguero-Rosenfeld M, Wormser GP (1997). Antigenic diversity of granulocytic *Ehrlichia* isolates from humans in Wisconsin and New York and a horse in California.. J Infect Dis.

[32] von Loewenich FD, Scorpio DG, Reischl U, Dumler JS, Bogdan C (2004). Control of *Anaplasma phagocytophilum*, an obligate intracellular pathogen, in the absence of inducible nitric oxide synthase, phagocyte NADPH oxidase, tumor necrosis factor, Toll-like receptor (TLR)2 and TLR4, or the TLR adaptor molecule MyD88.. Eur J Immunol.

[33] Parvanova I, Epiphanio S, Fauq A, Golde TE, Prudencio M (2009). A small molecule inhibitor of signal peptide peptidase inhibits *Plasmodium* development in the liver and decreases malaria severity.. PLoS One.

[34] Martens S, Sabel K, Lange R, Uthaiah R, Wolf E (2004). Mechanisms regulating the positioning of mouse p47 resistance GTPases LRG-47 and IIGP1 on cellular membranes: retargeting to plasma membrane induced by phagocytosis.. J Immunol.

[35] Tsuji M, Mattei D, Nussenzweig RS, Eichinger D, Zavala F (1994). Demonstration of heat-shock protein 70 in the sporozoite stage of malaria parasites.. Parasitol Res.

[36] Schaible UE, Kaufmann SH, Zychlinski A, Sansonnetti P (2002). Studying trafficking of intracellular pathogens in antigen presenting cells.. Methods in Microbiology.

[37] Martens S, Howard J (2006). The interferon-inducible GTPases.. Annu Rev Cell Dev Biol.

[38] Dunay IR, Heimesaat MM, Bushrab FN, Muller RH, Stocker H (2004). Atovaquone maintenance therapy prevents reactivation of toxoplasmic encephalitis in a murine model of reactivated toxoplasmosis.. Antimicrob Agents Chemother.

[39] Boehm U, Guethlein L, Klamp T, Ozbek K, Schaub A (1998). Two families of GTPases dominate the complex cellular response to IFN-gamma.. J Immunol.

[40] Zeng J, Parvanova IA, Howard JC (2009). A dedicated promoter drives constitutive expression of the cell-autonomous immune resistance GTPase, Irga6 (IIGP1) in mouse liver.. PLoS ONE.

[41] Prudencio M, Rodriguez A, Mota MM (2006). The silent path to thousands of merozoites: the *Plasmodium* liver stage. Nat Rev Microbiol..

[42] Coers J, Bernstein-Hanley I, Grotsky D, Parvanova I, Howard JC (2008). *Chlamydia muridarum* evades growth restriction by the IFN-gamma-inducible host resistance factor Irgb10.. J Immunol.

[43] Nelson DE, Virok DP, Wood H, Roshick C, Johnson RM (2005). Chlamydial IFN-gamma immune evasion is linked to host infection tropism.. Proc Natl Acad Sci U S A.

[44] Al-Zeer MA, Al-Younes HM, Braun PR, Zerrahn J, Meyer TF (2009). IFN-gamma-inducible Irga6 mediates host resistance against *Chlamydia trachomatis* via autophagy.. PLoS ONE.

[45] Zhao YO, Khaminets A, Hunn JP, Howard JC (2009). Disruption of the *Toxoplasma gondii* parasitophorous vacuole by IFNgamma-inducible immunity-related GTPases (IRG proteins) triggers necrotic cell death.. PLoS Pathog.

[46] Zhao Y, Ferguson DJ, Wilson DC, Howard JC, Sibley LD (2009). Virulent *Toxoplasma gondii* evade immunity-related GTPase-mediated parasite vacuole disruption within primed macrophages.. J Immunol.

[47] Fentress SJ, Behnke MS, Dunay IR, Mashayekhi M, Rommereim LM (2010). Phosphorylation of immunity-related GTPases by a *Toxoplasma gondii*-secreted kinase promotes macrophage survival and virulence.. Cell Host Microbe Dec.

[48] Steinfeldt T, Könen-Waisman S, Tong L, Pawlowski N, Lamkemeyer T (2010). Phosphorylation of IRG resistance proteins is an evasion strategy for virulent *T. gondii* strains.. PLoS Biology.

[49] Collazo CM, Yap GS, Sempowski GD, Lusby KC, Tessarollo L (2001). Inactivation of LRG-47 and IRG-47 reveals a family of interferon gamma-inducible genes with essential, pathogen-specific roles in resistance to infection.. J Exp Med.

[50] Hunn JP, Koenen-Waisman S, Papic N, Schroeder N, Pawlowski N (2008). Regulatory interactions between IRG resistance GTPases in the cellular response to *Toxoplasma gondii*.. Embo J.

[51] Coers J, Starnbach MN, Howard JC (2009). Modeling infectious disease in mice: co-adaptation and the role of host-specific IFNgamma responses.. PLoS Pathog.

[52] Zerrahn J, Schaible UE, Brinkmann V, Guhlich U, Kaufmann SH (2002). The IFN-inducible Golgi- and endoplasmic reticulum- associated 47-kDa GTPase IIGP is transiently expressed during listeriosis.. J Immunol.

[53] Hooper M, Hardy K, Handyside A, Hunter S, Monk M (1987). HPRT-deficient (Lesch-Nyhan) mouse embryos derived from germline colonization by cultured cells.. Nature.

[54] Schenten D, Gerlach VL, Guo C, Velasco-Miguel S, Hladik CL (2002). DNA polymerase kappa deficiency does not affect somatic hypermutation in mice.. Eur J Immunol.

[55] Kontgen F, Suss G, Stewart C, Steinmetz M, Bluethmann H (1993). Targeted disruption of the MHC class II Aa gene in C57BL/6 mice.. Int Immunol.

[56] Schwenk F, Baron U, Rajewsky K (1995). A cre-transgenic mouse strain for the ubiquitous deletion of loxP-flanked gene segments including deletion in germ cells.. Nucleic Acids Res.

