# Supplementary figures and images for: The IFN-γ-Inducible GTPase, Irga6, Protects Mice against Toxoplasma gondii but Not against Plasmodium berghei and Some Other Intracellular Pathogens
Source: PLoS One. 2011 Jun 17;6(6):e20568. doi: 10.1371/journal.pone.0020568 (PMC3117789; doi:10.1371/journal.pone.0020568)

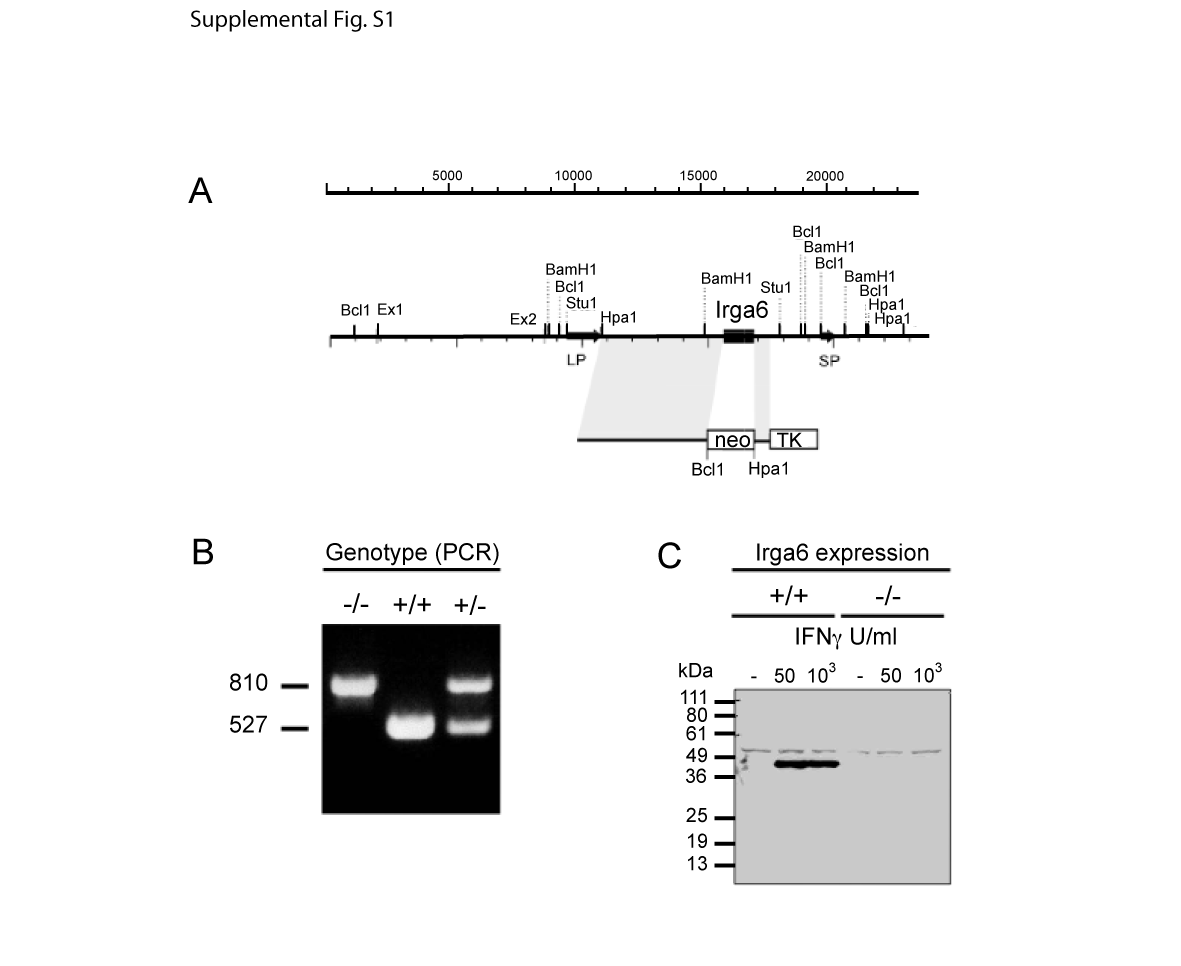

Supplement: Figure S1 — Generation of Irga6/IIGP gene deletion in Berlin. In the Max Planck Institute for Infection Biology, Berlin, Irga6/IIGP was originally cloned by suppression subtractive hybridization from splenocytes isolated from L. monocytogenes-infected C57BL/6 mice [52]. The following strategy was employed to generate a deletion of the Irga6/IIGP gene (Figure S1). LAWRIST7-based cosmids MPMGc121F04152 and MPMGc121E14552 from murine genomic 129/Ola cosmid library (Deutsches Ressourcenzentrum fuer Genomforschung RZPD, Berlin, Germany) were identified as Irga6/IIGP positive by PCR and confirmed by sequencing. A 4684 bp EcoRI/PvuII fragment, immediately upstream of the single coding exon of the Irga6 gene, was cloned in front of a neomycin resistance cassette within a pBluescript vector. A 590 bp PCR-amplified Irga6 fragment immediately downstream of the Irga6 coding sequence was cloned behind the selection cassette followed by a herpes simplex virus thymidine kinase cassette (Figure S1A). The linearised targeting vector was electroporated into E14.1 ES cells [53]. Homologous recombinants were detected by PCR and confirmed by Southern blot hybridization with 5′ or 3′ flanking probes. Single integration was verified by probing the Southern blots with the neomycin resistance cassette. Correctly targeted ES cell clones were injected into C57BL/6 blastocysts and transferred to foster mothers. Chimeric progeny were backcrossed either to C57BL/6 or 129Sv/J mice, and germ line transmission of the targeted allele was confirmed by Southern blot. The mice used in these experiments were backcrossed to the C57BL/6 background for at least 7 generations or to the 129/SvJ background for at least 4 generations. Genotyping for the Irga6 mutation was performed by PCR with the following primers: 5′- CTGCTGACCTAGTGAATATCATC -3′ (Irga6 forward), 5′- CGCCTTCTTGACGAGTTCTTCTG (Neomycin forward), and 5′- AATGTGGATACATAATCAGTAAAGG -3′(Irga6 reverse). The endogenous, non-mutated locus gave rise to a 527 bp fr [file pone.0020568.s001.tif]

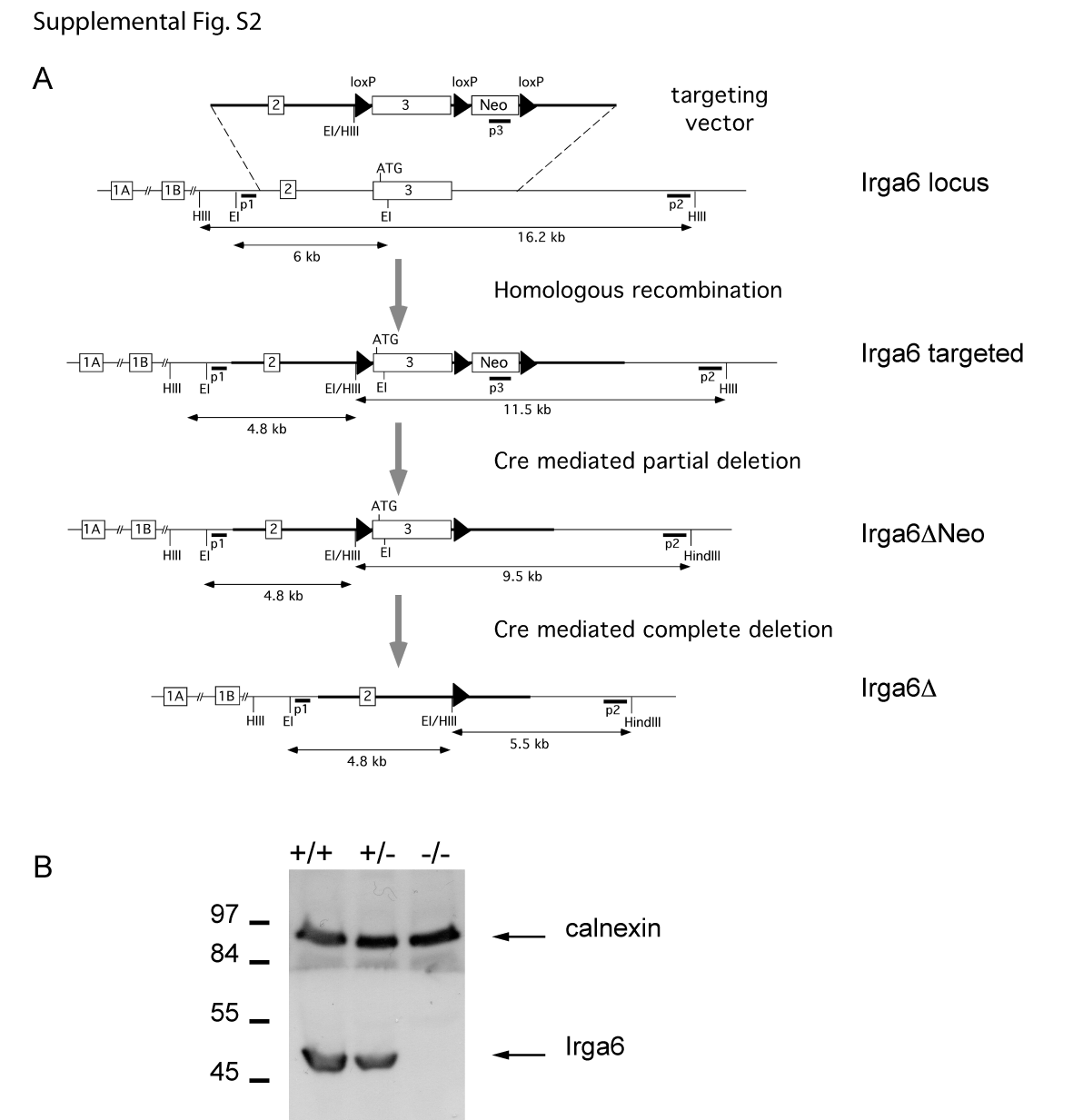

Supplement: Figure S2 — Generation of Irga6/IIGP gene deletion in Cologne. At the Institute for Genetics in Cologne, Irga6/IIGP was originally cloned from IFNγ-induced C57BL/6 mouse embryonic fibroblasts by suppression subtractive hybridization [39]. The following strategy was employed to generate a deletion of the Irga6/IIGP gene. A 4kb BamH1 fragment, containing the whole of the coding exon 2 of Irga6 with about 2 kb of 5′ intronic sequence and about 1 kb of 3′-untranslated sequence, was subcloned from the C57BL/6 strain derived genomic BAC clone RP23-19A12 into the blunted Sal1 site of the targeting vector, pEasyFlox [54] 5′ and 3′ homology arms were generated by PCR amplification from BAC sublones. Additional restriction sites were added with the primers to enable the 5′ and 3′ homology arms to be cloned into the Xho1 and Not1 sites of pEasyFlox respectively. The completed targeting construct (Figure S2A) was linearised and transfected into Bruce4 ES cells derived from C57BL/6 Thy1.1 mice [55]. Of 350 G418 and gancyclovir-resistant colonies, 3 were identified as homologous integrants, of which one was probably partial. The remaining two clones, 1B10 and 3A3 were injected into CB20 blastocysts and high-level chimeras generated. Two chimeras derived from clone 1B10 transmitted the mutant allele to 100% of progeny. A completely Irga6-deficient mouse was generated by crossing germ-line-transmitting offspring to the C57BL/6 Cre-deleter mouse [56] Positive heterozygous progeny were intercrossed to generate homozygous Irga6-deficient progeny. All expected genotypes were generated in mendelian ratios, assayed by Southern blotting of tail-tip DNA. Embryonic fibroblasts prepared from the homozygous line of Irga6-deficient mice showed no signal in Western blot with rabbit anti-Irga6 serum 24 hr after induction with 100 U/ml IFN-γ (Figure S2B). (TIF) [file pone.0020568.s002.tif]

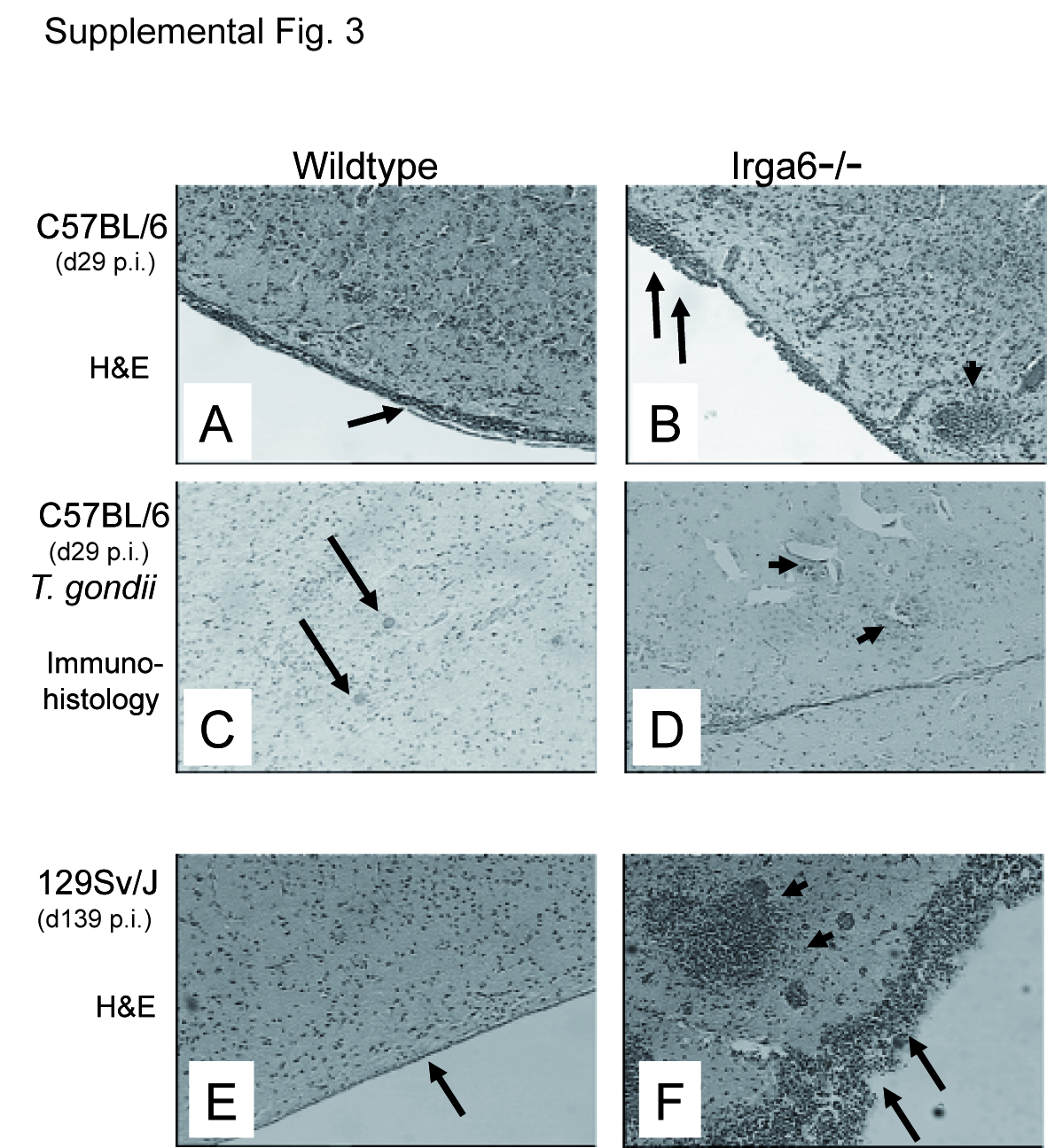

Supplement: Figure S3 — Histological changes in brains of wildtype and B-Irga6-deficient mice following infection with T. gondii . Histological changes in brains of mice infected orally with 10 cysts of the ME49 strain of T. gondii. At 29 (C57BL/6-background, A, B) and 139 (129Sv/J- background, E, F) days post infection brains of mice were obtained, sections prepared, and stained with H&E. Arrows in A, B, E, and F indicate meninges; arrowheads indicate areas of focal inflammation. To visualize parasites, sections of brains obtained at day 29 post infection were stained with an anti-T. gondii serum (C,D); T. gondii cysts are indicated by arrows and parasitophorous vacuoles are indicated by arrowheads. (TIF) [file pone.0020568.s003.tif]
